# Culturally adapting an evidence-based diabetes self-management education program for black adults: a pilot mixed methods implementation study

**DOI:** 10.3389/fpubh.2026.1806362

**Published:** 2026-08-04

**Authors:** Olubukola Tikare, Helen Omuya, Meng-Jung Wen, Tina Kansariwala, Annika Pickard, Janiya Pouncy, Martha Maurer, Olayinka O. Shiyanbola

**Affiliations:** 1 Department of Clinical Pharmacy, University of Michigan College of Pharmacy, Ann Arbor, MI, United States; 2 Division of Health Services Administration, University of Alabama, Birmingham, AL, United States; 3 Department of Public Health Sciences, University of Rochester, NY, United States; 4 Division of Social and Administrative Sciences, University of Wisconsin-Madison, Madison, WI, United States; 5 Department of Pharmacy Systems, Outcomes, and Policy, University of Illinois, Chicago, IL, United States

**Keywords:** black adults, cultural adapting, diabetes self-management, health disparities, implementation, mixed methods

## Abstract

**Introduction:**

Black adults in the United States disproportionately experience type 2 diabetes and related complications, yet participation in diabetes self-management education (DSME) is low. Culturally adapted approaches are needed to improve engagement and outcomes. This pilot study evaluated the implementation of a co-designed culturally adapted DSME program for Black adults using the RE-AIM framework and Proctor’s implementation outcomes.

**Design:**

This pilot mixed-methods implementation study evaluated a culturally adapted 6-week group based DSME program delivered in community settings. Thirty-two Black adults with diabetes or prediabetes were enrolled. Implementation outcomes included attendance, retention, and satisfaction. Participant-reported outcomes were assessed at baseline and 6 months, and qualitative interviews explored participant experiences.

**Results:**

Retention was 100%, with high attendance (86%) and strong satisfaction, indicating high feasibility and acceptability. Participants reported improvements in engagement, empowerment, and self-care behaviors. Qualitative findings highlighted the role of culturally affirming facilitation, trust and group support in promoting participation in DSME. Participants’ mean age was 57; 81% were female, and 75% had type 2 diabetes. Participants reported improvements in overall health (12.1%, *p* = 0.02), patient health engagement (82.8%, *p* < 0.01), and diabetes empowerment (19.7%, *p* < 0.01). Self-care behaviors, such as diet, blood glucose monitoring, and foot care, improved significantly. Retention was 100%, with average attendance of 86% and high satisfaction (mean 4.2/5). Qualitative findings highlighted empowerment, accountability, and trust, fostered by culturally affirming facilitation and group support. Participants recommended longer sessions and hybrid formats.

**Conclusion:**

The program was feasible and acceptable, and was associated with improvements in self-reported self-care and empowerment among Black adults. Findings support further testing in larger trials to assess long-term effectiveness and implementation. Given the single-arm design, findings should be interpreted as exploratory.

## Introduction

In the United States, the prevalence of type 2 diabetes (T2D) is higher among non-Hispanic Black adults compared to non-Hispanic whites (1). Also, non-Hispanic Black adults in the US experience disproportionately higher rates of complications and mortality from T2D compared to their non-Hispanic white counterparts (2). These diabetes related complications include macrovascular and microvascular complications, which can lead to amputation, kidney failure, and cardiovascular events (3).

There is strong evidence that Diabetes Self-Management Education (DSME) programs are effective in improving diabetes outcomes, including significant improvements in lifestyle behaviors and self-care practices, which lead to better clinical and health outcomes among individuals with T2D (4). A large-scale systematic review of 120 studies comparing outcomes for individuals with T2D who either did or did not participate in DSME found an association between DSME participation and clinically meaningful improvements in A1c (5).

DSME programs have the potential to improve outcomes for Black adults; however, significant gaps remain in participation and effectiveness within this population. A systematic review examining the impact of DSME on A1c levels among Black adults found that 56% of RCTs reported significant improvements in glycemic control after participating in a DSME program, indicating moderate overall effectiveness (6). Indeed, one meta-analysis found no significant effect of DSME on A1c among Black adults, suggesting it may be attributable to low program completion, recommending more rigorous approaches to cultural adaptations of DSME for Black adults (7).

US National Medicare and private insurance data indicate very low participation in DSME across all racial and ethnic groups (8). A large urban health system in the US found that within a year after diagnosis, nearly 4,000 people with diabetes were referred to DSME by their healthcare providers. Among this group, those who were Black and did not have a partner for support were least likely to participate, despite having improved access to healthcare insurance (9).

DSME is critical for optimal diabetes control, but many barriers deter Black adults from participating or completing DSME classes. To explore these barriers, in our prior study, we held semi-structured interviews and focus groups with Black adults who participated in DSME, Black facilitators, and other key stakeholders such as clinicians, certified diabetes care and education specialists, the organization that disseminated DSME (the purveyor), and community leaders from Black-serving organizations. The findings revealed several barriers to DSME participation among Black adults, including the lack of cultural adaptation of the program content, limited flexibility for facilitators to adapt or incorporate relevant material, and the observation that most facilitators leading the DSME classes in Black communities identified as white. Additionally, low community trust in the DSME program emerged as a significant obstacle to engagement.

Strategies proposed to increase the participation of Black adults included enhancing the cultural relevance of DSME content to improve engagement and increasing the number of Black facilitators to enhance the visibility and significance of DSME in Black communities (10). These findings underscore the critical need to build capacity among Black facilitators, providing the structure, guidance, and opportunities for them to tailor evidence-based DSME content using culturally relevant strategies and examples.

Studies have addressed these issues using culturally appropriate interventions that foster trust, empower community voices, and incorporate peer-led education (11). A systematic review and meta-analysis of 37 studies evaluated culturally adapted diabetes education programs, primarily conducted in the U.S. with African American and Latino participants. Most programs demonstrated improvements in diabetes knowledge, self-management behaviors, and clinical outcomes when compared to standard, non-adapted interventions. Among 20 randomized controlled trials (*n* = 3,094) included in the meta-analysis, culturally adapted programs were associated with a significant reduction in A1c levels (12). Wadi et al. (13) conducted a systematic review of 16 U.S.-based RCTs focusing on culturally adapted DSME interventions for adults of Black African descent. Approximately half of the participants demonstrated significant improvements in A1c levels within 6 to 8 months. The most effective interventions involved ethnically matched educators, were delivered in community settings, and included culturally relevant content. These results underscore the importance of culturally adapted DSME in enhancing glycemic control and engagement among Black populations (13).

Despite evidence that culturally adapted DSME improves clinical outcomes, engagement, and relevance for Black adults with diabetes, many existing studies have limitations that reduce their impact. Few studies have examined the implementation of culturally adapted DSME interventions for Black adults into real-world settings, leaving a gap in understanding how to effectively engage Black adults in diabetes management through sustainable, scalable models.

To address these gaps, this study aimed to evaluate the implementation (reach, adoption, and feasibility) of a culturally adapted DSME program for Black adults using the RE-AIM framework and Proctor’s implementation outcomes, and to explore signal of changes in participant-reported outcomes within the effectiveness domain. Specifically, we assessed reach, adoption, implementation, and exploratory changes in participant-reported outcomes in a community setting.

## Materials and methods

### Research design

An evidence-based DSME, Healthy Living with Diabetes (HLWD) curriculum, along with companion content, was used to deliver a culturally adapted diabetes self-management program for Black adults. The added content focused on integrating culturally appropriate information into the DSME and was co-designed by a Stakeholder Advisory Board. This study is a single-arm, six-month pilot mixed methods implementation study conducted in two community settings within a Midwestern city in the US that has a predominantly Black population.

#### Intervention description

The Healthy Living for You program is a culturally adapted version of the evidence based Healthy Living with Diabetes (HLWD) curriculum. The intervention consisted of a 6-week, group-based diabetes self-management education program delivered in community settings by trained facilitators. Sessions included education on diabetes management, healthy eating, physical activity, medication adherence, and self-monitoring.

Cultural adaptations were informed by a stakeholder advisory board and included culturally relevant content and examples, incorporation of participant lived experiences, and facilitation approaches that emphasized trust, open dialogue, and community engagement.

### Recruitment

Participants were eligible if they (1) self-identified as Black/African American, were over 18 years old, and (2) had type 1 diabetes, type 2 diabetes, gestational diabetes, or pre-diabetes.

Participants were recruited through community-based outreach strategies, including flyers, community organizations, and word-of-mouth referrals.

### Data collection

#### Quantitative measures

Using the four implementation domains of the RE-AIM framework – Reach, Effectiveness, Adoption, and Implementation—we adopted self-reported surveys and study coordination documents to collect data (14). Based on Reach, we assessed the recruitment and retention of participants of the two HLWD workshops. To evaluate the pre- and post-impact on diabetes-related outcomes for the Effectiveness domain of Re-AIM, self-reported questionnaires was collected before the intervention and six months after the intervention. The 25-min survey was administered in person at a community setting during participants’ data collection visits.

Participants’ adherence to specific self-care activities was measured using the summary of diabetes self-care activities scale (15). We also used the 5-item patient health engagement scale to assess participants’ multifaceted experiences with health management (16). The 10-item diabetes empowerment scale–short form was used to evaluate the psychosocial self-efficacy of individuals with diabetes (17). Patient-provider communication was measured using the 13-item patient’s perceived involvement in care scale (18). For Adoption, we evaluated facilitator adoption, facilitator satisfaction, and organizational adoption from program providers. To assess the feasibility of Implementation, we evaluated the fidelity of intervention delivery by facilitators, participant adherence, and fidelity of enactment. Participant demographic and diabetes-related clinical characteristics were also collected through self-reported surveys and study coordination documents.

#### Qualitative data

Thirty-two participants who participated in the Healthy Living for You, the 6-week culturally adapted diabetes self-management program, were interviewed using a semi-structured guide. Guided by the RE-AIM framework and Proctor’s implementation outcomes, we assessed the delivery of the program across reach, adoption, implementation feasibility, and participant–reported experiences related to the effectiveness and maintenance of the culturally adapted DSME intervention (14, 19, 20). The first interview collected feedback on participants’ experiences with the program delivery, explored what they learned from the program, and inquired about ways the program could be improved. This interview focused on recruitment, participant experiences with outcomes, adoption, and implementation. The second interview explored whether participants had continued their adopted lifestyle habits after the intervention. Participants reported maintaining lifestyle changes following the intervention and how they maintained the behavior changes. Implementation outcomes were mapped to RE-AIM domains, with reach assessed through recruitment and retention, adoption through facilitator uptake, and implementation through attendance and fidelity.

### Data analysis

#### Quantitative data

Descriptive statistics were used to evaluate participant demographic and clinical characteristics as well as implementation outcomes. Outcomes related to the possible impact of the intervention were measured at two time points: baseline and 6 months after the intervention ended. Within-group differences between pre- and post-intervention were analyzed using Wilcoxon Signed-Rank tests due to the small sample size and non-normal distribution of outcome variables.

**Figure 1:**
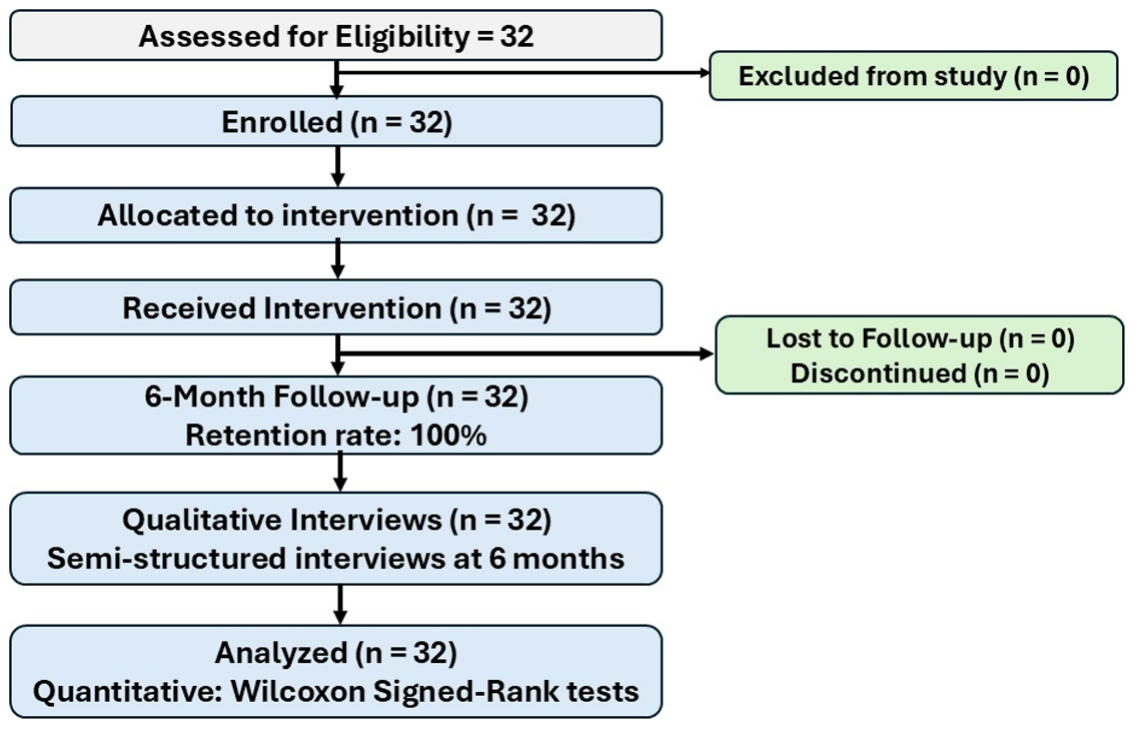
CONSORT flow diagram.

Given the single-arm design, analyses were exploratory and not intended to establish causal relationships between the intervention and observed outcomes. Within-group differences between pre-and post-intervention were analyzed using Wilcoxon Signed-Rank tests due to the small sample size and non-normal distribution of outcome variables. Because multiple exploratory outcomes comparisons were conducted, adjustments for multiplicity were not applied. Therefore, statistically significant findings should be treated as hypotheses-generating. Effect sizes for continuous outcomes were calculated as the standardized response mean, defined as the mean change from baseline to follow-up divided by the standard deviation of that change. SRM values of 0.2, 0.5 and 0.8 are conventionally interpreted as small, medium and large effects, respectively. Effect sizes were not calculated for binary outcomes; proportions and test statistics are reported instead, and this is noted in the table footnote. For binary pre-post outcomes, paired proportions were compared using McNemar tests when paired discordant counts were available. All statistical analyses were performed using IBM SPSS (version 28.0) (see Figure 1).

#### Qualitative data

After collecting the data and having it professionally transcribed, the research team used NVivo 15 to open-code the transcripts. The transcripts were first read repeatedly to achieve familiarization with the data. Meaningful words, phrases, and segments of participants’ narratives that reflected participants’ experiences and perceptions of the intervention were identified and assigned initial open codes. Similar codes were then compared and grouped into categories based on shared meaning and recurring patterns across participants. These categories were iteratively reviewed, refined, and synthesized into broader themes that captured the underlying concepts within the data. The coding and analysis were guided by Braun and Clarke’s steps to thematic analysis while incorporating RE-AIM framework domains (21). The analysts met to discuss the codes and themes and reached a consensus. A third member of the study team was invited to resolve discrepancies and ensure consistency in interpreting and developing the final themes.

#### Mixed methods data

An intervention mixed methods design was used in integrating quantitative data with qualitative to compare and generate implications for future improvement of the intervention. This approach was selected because quantitative data evaluated implementation and participant-reported outcomes, while qualitative data provided contextual insights into participants’ experiences and explanations for the observed findings, enabling a more comprehensive evaluation of the culturally adapted DSME program. We integrated qualitative data on participants’ experiences, including perceived benefits, challenges of participation, and feedback on intervention implementation, with quantitative findings. By comparing, integrating and merging data from both sources, we developed meta-inferences for the four RE-AIM domains: (1) Reach – implications for adopting new recruitment strategies, partners, adopters, or locations and identifying individual or structural facilitators and barriers to sustained program participation, (2) Effectiveness – insights into reasons for both positive and negative impacts of interventions, (3) Adoption – guidance for future modifications to program components, facilitator training, and implementation strategies to enhance adoption, and (4) Implementation – suggestions for improving program delivery, culturally adapted components, and intervention duration. Additionally, we adopted joint displays for each domain to present quantitative measures alongside qualitative summaries, allowing for clear visualization and reporting of the integration process.

#### Ethical statement

This mixed methods study was approved by the PI’s prior university’s Minimal Risk Research Institutional Review Board (Study ID: 2020-0821). This clinical trial is registered at: https://clinicaltrials.gov/study/NCT05917093.

## Results

### Participant demographics

The average age of participants (*n* = 32) was 57.1 years (SD = 12.1), and 81.2% were female. The mean number of chronic conditions was 3.3 (SD = 1.5), with an average duration of diabetes of 12.6 years (SD = 10.6). Participants reported taking an average of 3.3 medications (SD = 2.1) (Table 1).

**Table 1:** Demographic and clinical characteristics between groups at baseline.

Characteristics	*n* = 32	%
Age, mean (SD)	57.1 (± 12.1)	
Female	26	81.2%
Degree or above	20	62.5%
Household annual income < 20,000	17	53.1%
Condition		
Pre-diabetes	5	15.6%
Type 1 diabetes	2	6.3%
Type 2 diabetes	24	75.0%
Number of chronic conditions, mean (SD)	3.3 (±1.5)	
Years of diabetes diagnosis, mean (SD)	12.6 (± 10.6)	
Number of medicines taken, mean (SD)	3.3 (± 2.1)	

### Quantitative effectiveness outcomes

At six months, participants showed a statistically significant improvement in overall health (12.1% increase, *p* = 0.02, effect size = 0.48) and patient health engagement (82.8% increase, *p* < 0.01, effect size = 2.16). Diabetes empowerment also increased significantly by 19.7% (*p* < 0.01, effect size = 0.73). Patient-provider communication showed minimal change (1.6%, *p* = 0.89, effect size = 0.02), which was not statistically significant.

Signal of improvements were observed in self-management behaviors at six months following a healthy eating plan, following a specific diet, blood glucose monitoring, etc. No significant changes were found in medication-taking (*p* = 0.68) or smoking status (0%) (Table 2).

**Table 2:** Quantitative intervention outcomes.

Mean (SD) or *n* (%)	Expect	Baseline (T1)	6 months (T3)	T3–T1	Change (%)	Sig.	Effect size
Diabetes outcomes							
Overall health*	↑	2.64 (0.7)	2.96 (0.8)	0.32 (0.7)	12.1%	0.02	0.48
Patient health engagement**	↑	15.39 (3.6)	28.13 (7.6)	12.74 (5.9)	82.8%	<0.01	2.16
Emergency room visit	↓	10 (31.3%)	6 (21.4%)	−4	−40%	0.63	-^†^
Doctor visit for complications	↓	8 (30.8%)	2 (18.8%)	−6	−75%	0.53	-^†^
Diabetes empowerment**	↑	29.61 (7.7)	35.43 (7.0)	5.82 (7.9)	19.7%	<0.01	0.73
Patient–provider communication	↑	4.92 (3.1)	5.00 (3.3)	0.08 (3.4)	1.6%	0.89	0.02
Diabetes self-care activities							
Healthy eating plan*	↑	3.38 (2.2)	4.81 (1.5)	1.43 (2.9)	29.7%	0.01	0.49
Specific diet*	↑	3.63 (1.4)	4.90 (1.4)	1.28 (2.0)	35.3%	0.01	0.64
Exercise	↑	3.31 (2.2)	4.10 (2.3)	0.79 (2.6)	23.9%	0.12	0.37
Blood glucose monitoring*	↑	4.23 (2.8)	4.95 (3.0)	0.72 (2.2)	17.0%	0.04	0.33
Foot care*	↑	3.17 (2.5)	4.88 (2.1)	1.71 (2.7)	53.9%	0.01	0.65
Medication-taking	↑	5.96 (2.4)	6.39 (2.1)	0.43 (2.8)	7.2%	0.68	0.15
Smoking status	↓	9 (31.0%)	9 (31.0%)	—	0%	1.0	-

*p < 0.05, **p < 0.001.^†^Effect sizes are not reported for binary (yes/no) variables. These effect size is reported as the standardized response mean, calculated as mean paired change divided by the standard deviation of the paired change. These values describe the magnitude of within-person change in this pilot sample and should not be interpreted as causal effects.

### Quantitative implementation outcomes

Program implementation demonstrated strong feasibility and acceptability. Recruitment exceeded the target of 30 adults, with a recruitment rate of 107%, and all enrolled participants completed final data collection, resulting in a retention rate of 100%. Group session attendance averaged 86%, reflecting a high level of participant engagement. Overall satisfaction with the program was rated 4.2 out of 5.0. Among the program elements, facilitators received the highest rating (4.5/5.0), followed by the curriculum (4.3/5.0), workshop location (4.1/5.0), and workshop time (4.0/5.0).

Facilitator-related outcomes also indicated high feasibility of adoption. The facilitator adoption rate was 100%, and the mean satisfaction score for the training and facilitation experience was 4.3 out of 5.0. These findings suggest that the program was well-received by both participants and facilitators, supporting its potential for broader implementation (see Table 3).

**Table 3:** Joint display of participant qualitative and quantitative outcomes from the Healthy Living For You Intervention.

Quantitative data	Qualitative themes	Exemplar quotes (P=participant)	Meta-inferences
Reach			
Recruitment rate: 107% (32/30) Group session attendance: 86% Retention rate: 100% (32/32)	Multiple recruitment methods: using a public bulletin board to post flyers. words of mouth. community locations. social and audiovisual media. Barriers to participation included conflicts in schedule and difficulty comprehending some of the content in the learning materials. Willingness to attend future session: For shared experiences. To learn and re-educate oneself. To support family members.	I was referred by somebody in particular. And I’m a community health worker, so I was working under her organization. (P 4) Yeah, if it’s going to have new information because, okay, because as far as I’m concerned, I’m sticking to what I was taught in that class. (P 19) You know, at first, I was having a little issues, you know, where I was having to take my meds, …during the time, you know, when I started out. But, you know, I kind of rearranged my life a little bit, you know, where it worked out just fine.” (P 12)	*Expansion:* Participants shared how they heard about the program and ways to improve recruitment. They were able to overcome challenges to participation. They also explained what would make them attend a future program.
Effectiveness			
Adherence to diabetes self-care activities – blood glucose monitoring Mean score difference (%): increased 0.72 (+17.0%), *p* < 0.01 Effect size: 0.33	Steps taken to monitor or maintain appropriate blood sugar levels. Action plan related to monitoring glucose values. Actively checks blood sugar. Developing action plans served as motivation to manage diabetes. Program was informative on how to check blood sugar. Developed awareness of medicine and glucose values.	“… I got a whole lot of tests just, you know, that I have not used… And now I more consciously checking them more often… this class, you know…kind of did it for me as far as knowing where you are as far as your numbers go.” (P 8)	*Confirmation:* Participants in this study report monitoring their blood sugar more consistently. *Expansion:* Participants perceived the program improved their knowledge on monitoring and maintaining an ideal blood glucose level by informing them of the importance and need to consistently check and know their readings.
Diabetes outcomes—patient health engagement Mean score difference (%): increased 12.7 (+82.8%), *p* < 0.01 Effect size: 2.16	Participants recruited in this study are able to asks questions from healthcare providers about health issues. Learned to use strategies on how to communicate with healthcare providers. Understand what questions to ask their healthcare providers. Increased trust in healthcare providers and comfortability to share issues. Learned about what devices and medications should be consulted with healthcare providers.	“They kind of said that, you know, we are our own advocates for our own bodies and that, you know, if the medical or the doctor or the medical institution that we go to is not helping us in any type of way or is not doing what we would like for them to do after repeated attempts, then we can say, hey, you know, I do not work for you. You work for me… So now my doctor is really good, and she’s treating me good, you know, for so far… So I thought that that was really good that they gave me a voice, you know. So that was something that I got out of it is a voice (P 18). “If I have questions, talk to the doctor about them, because I have an opinion also. The doctor knows best, but I can also speak up for myself, and if I have questions before I get to the doctor, write them down and take them with me and discuss it.” (P 30)	*Confirmation:* Program increased participants’ engagement with their healthcare provider by making them better advocates for their healthcare needs. *Expansion:* By building trust and initiating questions, participants could communicate more effectively with healthcare providers and better advocate for their healthcare needs.
Diabetes outcomes—overall health Mean score difference (%): increased 0.32 (+12.1%), *p* < 0.05 Effect size: 0.48	Overall changes to daily activities to improve overall health. Increased exercise and walking Yoga and Meditation Lifestyle consciousness	“I’ve tried to increase my time for exercising because I do have a gym membership through my AARP, not AARP, my United Healthcare. And depending on how my knee is, I try and go at least three times a week.” P 19 “I’ve gotten more serious about my lifestyle with diabetes, and the program helped me to do that.” P 31	*Confirmation:* Program motivated participants to improve healthy behaviors which lead to an improvement in overall health.
Diabetes outcomes - diabetes empowerment Mean score difference (%): increased 5.82 (+19.7%), *p* < 0.01 Effect size: 0.73	Empowering and culturally relevant facilitation for inclusive and engaging diabetes management. Facilitators created a safe place for sharing. ◦ Non-judgmental learning environment. ◦ Opportunities to share stories of managing diabetes with others. Facilitators made the sessions to be engaging for all participants. Facilitators: ◦ Encouraged them by discussing the relationships between self and illness. ◦ Encouraged participants to stick to their action plan.	“So it helped me deal, it helped me learn, have tools to deal better with those things that are going to come up, obstacles that’s going to come up. And so I do not get as upset, angry, sad, and discouraged as much as I used to.” (P 24). “To be honest with you, I’m a single mother, and I live with my parents that are dealing with diabetes, very high diabetes. And when I saw them, I just, you know, when I see them, how they are living and their condition, that does not give me a time to relax when it comes to diabetes. So I have to keep up on top of my game as far as taking care of myself and managing my blood sugar so I could be help for parents, my kids…” (P 32)	*Expansion:* Participants were empowered in their diabetes selfcare by being better able to manage stress and be proactive about their selfcare.
Adherence to diabetes self-care activities – healthy eating plan mean score difference (%): increased 1.43 (+29.7%), *p* < 0.01 Effect size: 0.49	Healthy Eating Checking and counting calories Eat right Limit sweets Portion control Reading food labels	“So talking with and listening to some of the other participants and the facilitators gave me more ideas on things and that I could cook or change the way I cook and adding other ingredients to make the spices and such that would make the food taste better towards some things that were healthier that I did not like to eat.” (P 29) “I started to drink more water… I’ve eaten more vegetables and more fruits for my dinner hour. I do two meals a day instead of three, and I’m exercising more. I did not pick up any weight, and I did not lose any weight. But that’s… about to come next.” (P 13) “I get a little, like in the middle of the day, I take my little snack. I eat little portions of that. I eat peanuts. I eat a lot of vegetables. I eat a lot of fruit. And I eat that, then I got portions of that.” (P 2)	*Confirmation:* Participants were able to learn what constitutes a healthy meal and how to make healthy meals. This led to an improvement in their ability to plan healthier meals. *Expansion:* By learning to plan for healthier meals, participants were able to improve their weight management.
Adherence to diabetes self-care activities – foot care Mean score difference (%): increased 1.71 (+53.9%), *p* < 0.01 Effect size: 0.65	Feet Care Checking feet regularly Seeking better feet care	“Oh, since the workshop, I’ve asked for like, I’m trying to get a better podiatrist. … And I’m afraid not, to have anybody just cutting on my feet or cutting my nails. I need somebody that knows what they are doing… That’s my main concern is my foot care.” (P 10). “When I went to the doctor after this program, it was just ringing in my head, take off your shoes, you know, and let them see your feet, you know, that type of thing, you know.” (P 8)	*Confirmation:* Participants had an increased awareness of the need to take care of their feet by seeking better professional care for their feet. *Expansion:* By learning how to take care of their feet, participants could ask their health care providers to examine their feet for any concern during an office visit.
Implementation			
Weekly session attendance: 86%	Participants and facilitators appreciated the cultural similarity. Barriers to participation included conflicts in schedule and content heavy sessions.	“I found it very refreshing to have facilitators who look like me, who understood culturally how I eat and gave suggestions on how to incorporate certain things that we eat culturally into my plan instead of me feeling.” (P 5)	*Confirmation:* Culturally adapted content, dedicated time to establish group norms and create a safe place for sharing, encouraged participation *Expansion:* Consider providing virtual options for participation.
Participant program acceptability Overall satisfaction = 4.2/5.0 Curriculum (4.3), facilitators (4.5), workshop time (4.0), workshop location (4.1)	Culturally relevant and empowering facilitation created safe space, an engaging environment. Valued facilitators’ relatable, personalized interactions, and cultural alignment. Enhancing participant engagement and support through adapted program modifications Redesign content delivery opinions on workshop timing: Program session times conflicted with personal schedule Sessions should have been longer Sessions were late in terms of scheduling Implement virtual sessions to reduce timing and travel conflicts Suggested including facilitators with research knowledge.	“… the instructors, they were very nice… up front, honest. They told us about their lives… experiences with people in their families with diabetes and, you know, other health issues.” (P 18) “I did not really like the flyers that would hang up because I could not see them. And plus I was in a like everybody wanted to say something. And you did not have a lot of time to just make sure, you know, you got that handout, the materials, and you went over some things, pointed out some stuff. I did not get a lot of that part. But I read my book, and I like the way the book explains.” (P 10) “I kind of hate the class ending so fast. I was like, it’s 6:00 already? It just went so fast, but it was just so much knowledge and everything.” (P 24) “We would have preferred somebody that knows more about the, how it works. The educator… should know more. The workshop, it did not tell us more about the research, and everything they were saying was in the book. The book was excellent.” (P 32)	*Confirmation:* Black adults demonstrated high satisfaction and acceptability. *Discordance:* Some participants expressed that session was content heavy and they were not able to catch up with the material in real time. Participants also shared that the time for the sessions was short and the curriculum could be improved by including diabetics research professionals and information from research studies.

### Qualitative results

#### Recruitment

##### Clear information to create awareness about diabetes

Participants appreciated the clear and informative recruitment process, which helped build trust and encouraged participation. Participants emphasized the importance of using a variety of outreach techniques including radio commercials, flyers, community centers, and word of mouth. Sharing information that was easy to understand and relevant through many channels helped people get involved.

“*The recruitment was very enlightening, very informative. Everyone that was there to teach us answered every question. They even added questions, modified*.” – Participant 3

“*Because I was referred by somebody in particular. And I’m a community health worker, so I was working under her organization. And she, I told her that I was a diabetic, and she said, here’s an opportunity for you. And I knew in the past that people get paid for research studies, because they advertise them on social media and stuff like that. So I wasn’t going to do it, but I just took the opportunity to say, you know, well, why not? And that’s how I got recruited.*” – Participant 4

#### Effectiveness

##### Diabetes empowerment through personal commitment, community and support and education

Participants discussed how their innate drive to control their diabetes, often shaped by personal, or family medical histories led to a sense of empowerment. The program’s education raised their health consciousness, encouraged preventative health practices, and changed their long-held assumptions about diabetes and medicine. Their dedication to healthy behaviors was strengthened by the community’s support, which came from social incentives, accountability, and shared experiences. Collectively, these components gave participants greater self-assurance, knowledge, and involvement in controlling their illness and standing up for themselves in medical environments.

“*Because of my family history, there could be a potential for me too in a pre-diabetes, diabetes. So I want to learn about it more for myself as well and just to be in the know, because some of the things I learned, I did not know that it was, could be affecting my brother. So now that I learned, I can do better, and therefore he can do better*.” – Participant 1.

“*My experience was great because I really think I really came out with a little bit of improvement, like stop eating sweets, and I start exercising more, and I started watching my diet. So and I started checking my sugars more, try to keep a track on my sugar levels and start exercising more. So I think I learned a lot from the class*.” – Participant 17

##### Learned about diabetes strategies in maintaining healthy diet, communicating with healthcare providers, managing complications

Participants expressed that they learned various diabetes management techniques through the classes. Verbatim statements included:

“*I was already trying to eat healthier because my liver doctor wanted me to lose some weight. So I had changed some things. So talking with and listening to some of the other participants and the facilitators gave me more ideas on things and that I could cook or change the way I cook and adding other ingredients to make the spices and such that would make the food taste better towards some things that were healthier that I did not like to eat*.” – Participant 29

“… I *read the book right now in detail. Now I understand why I’m having a muscle spasm, because I stopped walking for a while and then all of the sudden, I go back how I used to do. That’s why I have the muscle spasms. But in the book, it says that when you stop exercising, working out, then slowly start, slowly, not as you used to, because the muscle almost forgot everything. So that’s very, very helpful*.” – Participant 32

#### Intervention adherence

##### Action plans for diabetes self-management allowed participants to hold themselves accountable

Participants expressed how they were able to take charge of their diabetes care by developing personalized action plans that focused on blood glucose monitoring, exercise, healthy eating, medication adherence, and sleep. These plans strengthened an attitude of accountability and assisted in breaking goals down into achievable steps. They experienced obstacles like transportation, physical constraints, and conflicting life demands, even though many people found the structure inspiring and useful. However, in order to transform these action plans into long-lasting habits, participants underlined the importance of support networks, mental adjustments, and ongoing practice.

“*Mine was walking, how I’m going to exercise. So I was able to be successful with that, especially when they said you can just break it up in little, bite-size pieces. As long as at the end of the day you have done an hour, you have satisfied that, your action plan. So that’s what helped me*.” – Participant 1

“*I’m sorry to say, well, it helped me to accept the fact that I have diabetes now, and then I can check on my sugars daily. I can eat with the, eat more, how can I put it, eat healthier*.” –Participant 17

#### Participant acceptability

##### Empowering and culturally relevant facilitation for inclusive and engaging diabetes management

Participants reported positive perspectives on how facilitators created an inclusive and safe environment for sharing experiences in managing diabetes. Participants described the facilitators as central to creating a respectful, culturally affirming, and inclusive learning environment that fostered engagement and openness. Facilitators used relatable language, encouraged dialogue, and preserved confidentiality. Their culturally relevant teaching styles such as adapting recipes, acknowledging shared lived experiences, and understanding sociocultural norms deepened participant connection and trust. The program’s ability to reflect participants’ identities and experiences, alongside logistical support like transportation and meals, enhanced accessibility and sustained motivation. Many expressed a desire to re-attend, recommend the program, or even become facilitators themselves.

“*It was very engaging. They treated us like adults. They were very respectful, and the class was run very well. The two women, they looked like they got along very well. They were able to navigate the class very, very well. And, like I said, they were able to give us visuals in writing the information on the board. That was really good*.” – Participant 11

“*Everybody came in agreement and had an understanding, that we’d share, we’d listen to each other, and, share your ideas and share your thoughts. And, nobody is, judging. Everybody is different. Everybody goes through different things. Everybody deals with this disease differently. So …they made us feel like we had a safe space to talk about the things that we were going through, and then gave information, shared personal stories and everything like that, just brought it all together really well*.” – Participant 7

#### Implementation

##### Balancing in-person engagement with flexible options and time management for optimal learning

Participants appreciated the in-person format because it enabled them to connect with one another, learn from each other, and foster a supportive group dynamic. A wide range of participants believed that meeting in person made them more involved and helped to make the program feel like a “family.” However, they also noted time-related challenges such as long days, rushed arrivals, and limited discussion time. A few suggestions for improvement were to offer flexible scheduling, add virtual or hybrid options, and utilize multimedia tools such as videos or electronic summaries to enhance learning. Participants also suggested expanding support, offering localized services, and forming partnerships with community resources to make the program more engaging and accessible.

“*You got to interact with the other people in the group, and you got to see, to talk to them, and hear their genuine and, you know, it’s different than talking online than it is in person. I think in, talking in person is more personal*.” – Participant 19

“*Oh, I like in person because you get to meet the people who are your class, and you become like a little family. You laugh and talk, and that was so great. We all got along with each other, so that was one thing about in person, everybody gets along*.” – Participant 17

By integrating the results from the quantitative assessment with insights from qualitative interviews, we gained a more comprehensive understanding of the program’s impact on study participants. The quantitative data provided measurable outcomes in recruitment and retention, indicating signal of changes in behavioral measures. Several factors contributed to participants’ satisfaction, attendance, and acceptance of the intervention. Participants reported a greater sense of empowerment and ownership over their diabetes management. Although the quantitative data showed a high overall acceptance, participants suggested opportunities for improvement in the curriculum and intervention design. The meta inference highlighted the program’s acceptance and signal of effectiveness in providing Black adults with diabetes among study participants with the knowledge and confidence necessary to manage their diabetes in the long term.

## Discussion

This study found that Healthy Living for You, our culturally adapted DSME intervention, was both feasible and well-received by Black participants managing diabetes. Attendance at group sessions was notably high at 86%, which is significant considering that earlier DSME efforts aimed at Black populations often faced attendance issues caused by barriers such as a lack of cultural relevance. The strong attendance suggests that the program’s structure, content, and delivery effectively met the needs and expectations of the participants.

Participants also reported a high level of satisfaction with the program with facilitators receiving the highest scores (4.5 out of 5). Satisfaction is a marker of a well-liked program and can influence sustained engagement, follow-through on behavior change, and trust in the diabetes education being provided (22). Many of the program participants reported feeling respected, heard, and supported during the sessions, which likely contributed to their continued participation.

Participants reported improvements in health-related outcomes, including overall health, patient engagement, and diabetes empowerment. These findings may reflect improvements in knowledge, confidence, and motivation to manage diabetes more proactively. These outcomes reflect improved knowledge and heightened confidence with motivation to manage diabetes more proactively. Participants reported improvements in self-care activities, including healthier eating habits, better adherence to dietary plans, more consistent blood sugar monitoring, and increased attention to foot care. These self-management practices are essential for avoiding complications and enhancing long-term outcomes for individuals living with diabetes (23, 24).

The qualitative feedback from participants provided essential insight into why the program was well-received and why attendance remained high. Many participants mentioned the facilitators’ culturally affirming approach, including listening without judgment, using relatable examples, and acknowledging their lived experiences, helped them feel seen, respected, and motivated. These culturally adapted elements may be critical for the implementation of DSME programs targeting Black adults, as low participation and engagement have long been documented challenges (6).

Implementation outcomes, including attendance, satisfaction, and engagement, are important indicators of program uptake and may support participant engagement with diabetes self-management behaviors. The more consistent individuals attend and participate in DSME, the more likely they are to engage in key diabetes management behaviors, apply self-care strategies, and build the confidence needed to manage their diabetes. In this case, the program’s cultural responsiveness may have fostered an environment where participants felt safe to ask questions, share personal challenges, and take ownership of their health, ultimately enhancing the impact of the intervention.

By focusing on participants lived experiences and fostering trust, this program appeared to enhance the delivery of DSME and increase its receptivity among Black adults. These findings support a growing body of evidence that culturally adapted DSME can serve as an effective tool to reduce gaps in diabetes outcomes and provide a more equitable model of care (25, 26).

Beyond successful implementation, participants’ reported improvements in health and diabetes self-care behaviors. These changes varied from perceived enhancements in overall health to more consistent adherence to healthy eating plans, glucose monitoring, and foot care, indicating that the cultural adaptation of the DSME may have contributed to fostering behavior change. Several participants explained how the relatable teaching style, open group discussions, and respect for individual experiences helped build both confidence and accountability. Feeling understood and supported appeared to translate into increased motivation to apply what they learned outside of the group setting.

These findings support previous studies showing that culturally adapted DSME programs are associated with improved engagement and behavior change (27, 28). In contrast to generic models, culturally responsive programs effectively address prevalent barriers, such as mistrust, complex medical language, and stigma. These issues can hinder complete involvement in conventional DSME. Here, the program not only provided essential diabetes education but also did so in a manner that validated the identities and experiences of participants, potentially fostering a more robust foundation for enduring behavior change.

While many outcomes showed positive change, not all measures improved significantly. For example, patient-provider communication did not demonstrate a meaningful change over the course of the program. This was somewhat unexpected, given that participants reported feeling more confident and empowered in managing their diabetes overall. However, the qualitative interviews offered insights that may help explain this result. Several participants shared that although they had learned more about their condition and felt better equipped to manage it, their interactions with healthcare providers remained constrained by short appointment times, communication barriers, or a perceived lack of cultural understanding from their clinicians. It is possible that though the program seemed to enhance participants’ willingness to engage, it may not have sufficiently tackled the underlying structural or relational challenges within the healthcare system. Some participants expressed that they continued to feel hurried during medical appointments or reluctant to voice their concerns, even as they gained confidence in group environments. These experiences underscore the necessity for wider systemic initiatives to train providers in culturally responsive care and increase access to relationship-centered primary care alongside support interventions directed at patients. This gap between internal empowerment and external communication underscores the challenges of improving diabetes outcomes in marginalized communities, suggesting that strengthening patient-provider relationships requires more than just education, but also involves fostering trust, continuity, and a supportive environment for patients within clinical settings.

These findings highlight the potential of culturally adapted DSME programs and emphasize the importance of conducting a rigorously powered RCT randomized control trial to rigorously evaluate effectiveness. A larger study with an extended follow-up could clarify whether the observed improvements in health behaviors and patient engagement lead to sustained glycemic control, fewer complications, and decreased healthcare utilization. It would also allow for more detailed subgroup analyses to examine how factors such as gender, comorbidities, or initial empowerment influence outcomes.

Within practice settings, clinicians should build trust and engage patients by acknowledging their unique experiences. Using culturally appropriate approaches, such as involving trusted facilitators, holding group visits, or partnering with community organizations, can create more equitable paths for diabetes self-management and improve outcomes among traditionally and historically marginalized populations.

Policymakers also have a crucial role. As diabetes prevalence and disease complications rise, especially in Black communities, investing in scalable, community-based interventions that address both medical and social determinants of health becomes urgent. Programs like Healthy Living For You, a culturally adapted DSME program demonstrates that culturally responsive and empowering education may enhance participation and is associated with improved engagement in self-management programs and improved outcomes. Supporting such initiatives through program funding and training of culturally matched community facilitators can help reduce persistent disparities in diabetes care and outcomes.

This study has several important strengths. The use of an intervention mixed-methods design allowed for a more nuanced understanding of both the possible effectiveness and implementation of the intervention. Quantitative findings were enriched by qualitative insights that captured participants’ personal experiences, motivations, and perceived barriers, providing depth that would not have been possible through surveys alone. In addition, applying implementation science frameworks helped structure the evaluation to emphasize both outcomes and context, making the findings more actionable for future scale-up. A key strength of this study is the use of an initial co-design phase with Black adults and DSME facilitators to inform program development prior to implementation. Rather than applying post-hoc cultural adaptations, this study embedded community-informed input at the earliest stages of intervention design, ensuring that program content, delivery strategies, and facilitator guidance reflected the lived experiences and contextual needs of Black adults.

However, a few limitations should be acknowledged. As a single-arm study without a control group, we cannot attribute observed changes to the intervention alone. Also, we did not measure clinical outcomes such as A1C, blood pressure, or lipid levels, which are commonly used in diabetes studies to assess disease control. While improvements in self-reported behaviors are encouraging, future research should incorporate physiological outcomes to directly evaluate the intervention’s clinical impact. Although the brief follow-up period limits our ability to draw conclusions about long-term effects, the noted improvements suggest a possible positive influence. Despite these limitations, the consistent patterns observed across both qualitative and quantitative data suggest a preliminary signal of change that warrants further evaluation in a fully powered randomized controlled trial. This study also involved multiple exploratory comparisons without adjustment for multiplicity, increasing the possibility of type I error. Therefore, findings should be interpreted as exploratory and warrant confirmation in future controlled studies.

## Conclusion

This pilot study provides preliminary evidence that a culturally adapted diabetes self-management education program can be both acceptable and effective for Black adults living with diabetes, addressing participation and engagement challenges in these programs. Participant reported improvements in engagement, self-care behaviors, and perceived health, along with high satisfaction and attendance, suggest the potential for broader implementation. However, given the single-arm design and reliance on self-reported outcomes, these findings should be interpreted as exploratory. These findings establish a crucial foundation for a fully powered hybrid dissemination and implementation randomized controlled trial, involving a larger sample, extended follow-up, and the inclusion of clinical outcomes. Such a trial would enable more rigorous evaluation of the program’s impact and scalability and could significantly contribute to efforts aimed at reducing ongoing disparities in diabetes outcomes.

## Data Availability

The raw data supporting the conclusions of this article may be made available by the authors, upon request.
